# An exploratory human study of superstable homogeneous lipiodol–indocyanine green formulation for precise surgical navigation in liver cancer

**DOI:** 10.1002/btm2.10404

**Published:** 2022-09-10

**Authors:** Pan He, Yongfu Xiong, Bin Luo, Jianming Liu, Yang Zhang, Yu Xiong, Song Su, Cheng Fang, Yisheng Peng, Hongwei Cheng, Chengchao Chu, Jingsong Mao, Jingdong Li, Bo Li, Zhenyu Yin, Jie Tian, Gang Liu

**Affiliations:** ^1^ State Key Laboratory of Molecular Vaccinology and Molecular Diagnostics, Center for Molecular Imaging and Translational Medicine, School of Public Health Xiamen University Xiamen China; ^2^ Department of Hepatobiliary Surgery, Academician (Expert) Workstation Affiliated Hospital of North Sichuan Medical College Nanchong China; ^3^ Department of Hepatobiliary Surgery Affiliated Hospital of Southwest Medical University Luzhou China; ^4^ Department of Hepatobiliary Surgery Zhong'shan Hospital of Xiamen University Xiamen China; ^5^ Amoy Hopeful Biotechnology Co., Ltd. Xiamen China; ^6^ Key Laboratory of Molecular Imaging, Institute of Automation Chinese Academy of Sciences Beijing China

**Keywords:** conversion therapy, fluorescence imaging, hepatectomy, indocyanine green, lipiodol

## Abstract

The clinical applications of transcatheter arterial embolization (TAE) conversion therapy combined with hepatectomy have been severely restricted by ill‐defined tumoral boundaries and miniscule hidden lesions. Fluorescent surgical navigation is a promising method for overcoming these barriers. However, sufficient delivery of the fluorescent probe into the tumor region after long‐term TAE is challenging due to blockade of the tumor‐supplying artery. Here, a super‐stable homogeneous intermix formulating technology (SHIFT) to physically mix lipiodol and indocyanine green (ICG) formulation (SHIFT and ICG) for fluorescent surgical navigation after long‐term TAE conversion therapy is provided. Through the retrospective study of 45 clinical liver cancer patients, it is found that SHIFT and ICG formulation have excellent tumor deposition effect and safety. During surgical resection after long‐term TAE conversion therapy, SHIFT and ICG could clearly identify in real time the full tumor regions and boundaries and had a high signal‐to‐normal tissues ratio—even the indistinguishable satellite lesions could be identified with a strong fluorescence intensity. Meanwhile, SHIFT and ICG could improve operative, anesthetic, and postoperative variables associated with postoperative complications. This simple and effective SHIFT could provide precise fluorescent navigation for surgical resection following long‐term embolization therapy in clinical practice and has great potential for a translational pipeline.

## 1 INTRODUCTION

Liver cancer, the sixth most prevalent malignancy worldwide, is characterized by poor prognosis and high mortality rates.^1^ Radical resection remains the first line of treatment for liver cancer.^2^ However, due to the disease's rapid progression, many patients lost the opportunity for initial surgical resection when seeking treatment. Direct resection in cases in which there is a large tumor volume or a heavy tumor burden can cause excessive trauma, while the insufficient residual liver volume may lead to postoperative liver failure.^3^, ^4^, ^5^ Therefore, to prolong relapse‐free and overall survival, it is essential to improve the overall prognosis of liver cancer by transforming initially unresectable patients into operable patients, palliative surgery into a radical resection, and the R1 resection into an R0 resection.^6^, ^7^

The concept of conversion therapy was first proposed in 1977 by Shafer and Selinkoff.^8^ Transcatheter arterial embolization (TAE) is the most common method of conversion therapy; it embolizes the tumor's blood supply arteries to control tumor growth and reduces the proliferation of cancer cells caused by intraoperative extrusion for the improvement of radical surgical resection rate.^9^, ^10^, ^11^ However, during surgical resection, identifying small lesions and tumor boundaries is often difficult, leading to residual liver cancer tissues and postoperative tumor recurrence,^12^, ^13^ insufficient residual liver volume, and a hindered recovery of liver function after hepatectomy.^14^ Therefore, molecular imaging must be developed to distinguish boundaries and small tumor lesions for accurate complete resection of tumor lesions with minimal trauma.

Indocyanine green (ICG) is a near‐infrared fluorescent dye. Given its excellent optical properties and biosafety in vivo, ICG has been used in fluorescence‐guided surgical resection and intraoperative tumor visualization.^15^, ^16^, ^17^ However, in the interventional embolization of conversion therapy for liver cancer, in cases in which ICG—a small molecule—is injected intravenously (*i.v*.) prior to tumor embolization, the limited stability, ease of metabolism, and agglomeration quenching issues of ICG lead to poor fluorescence performance.^18^ Administration of *i.v*. ICG after tumor embolization prevents the infiltration and distribution of ICG in the tumor tissues that results from the blocked supply artery and tumor necrosis.^19^, ^20^ Therefore, surgical resection after interventional embolization of conversion therapy cannot be effectively and precisely navigated in the clinic.

**FIGURE 1:**
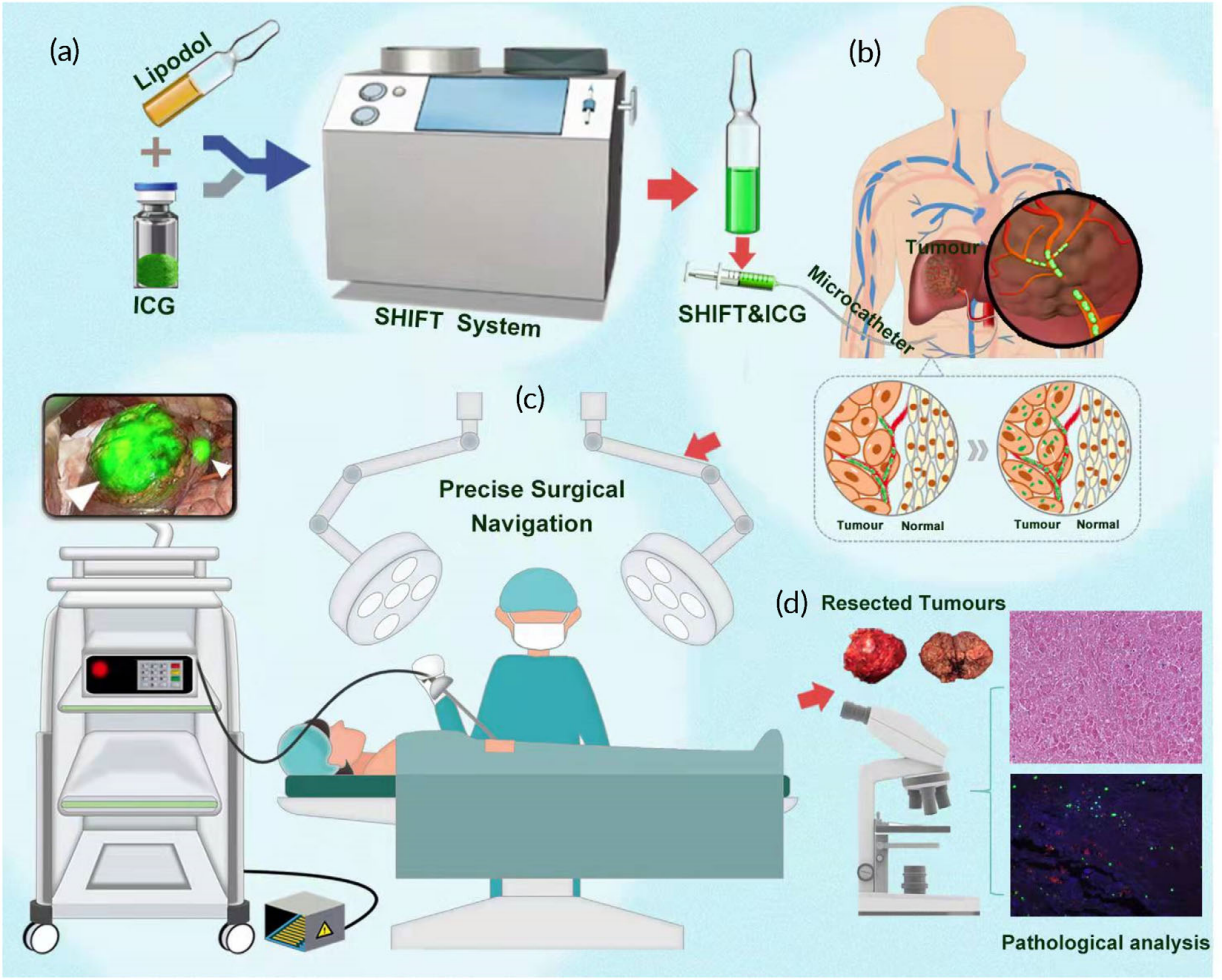
Schematic illustration of super‐stable homogeneous intermix formulating technology (SHIFT) and indocyanine green (ICG) preparation and research process. First, (a) superstable homogeneous intermix formulation system was employed to produce SHIFT and ICG with enhanced imaging properties, anti‐photobleaching capacity, and retention ability in tumor regions. Next, (b) liver cancer patients unable to be radically resected were enrolled in the study and received transcatheter arterial embolization (TAE) treatment with SHIFT and ICG as the main embolic agent. Subsequently, (c) the patients who reached the standard of radical surgical resection after TAE conversion therapy received a precise hepatectomy under real‐time fluorescence. (d) After the operation, pathological examination of the resected tissues was conducted.

To address this issue, we developed a super‐stable homogeneous intermix formulating technology (SHIFT) to physically mix lipiodol and ICG formulations (SHIFT and ICG) homogeneously for fluorescence‐navigated hepatectomy after long‐term TAE conversion therapy, which realized the interventional embolization with one‐time administration and follow‐up surgical fluorescence navigation. Previous work has confirmed that SHIFT formulation was prepared in a green physical mixture via a carrier‐free manner, which possesses controlled morphology, long‐term stability, and improved optical characteristics of ICG. Furthermore, the viscosity of the SHIFT and ICG was comparable to lipiodol and exhibited long‐lasting excellent fluorescence navigation in VX2 orthotopic hepatocarcinoma models.^21^ To elaborate on the results of the superior performance of SHIFT and ICG previously reported,^22^, ^23^ we conducted a clinical application trial. The SHIFT and ICG formulation prepared via SHIFT performs excellent tumor‐specific deposition after long‐term TAE therapy, which enables ICG to continuously illuminate the tumor regions and microsatellite lesions for specific resection (Figure 1). SHIFT and ICG assist surgeons at distinguishing proper lesions and determining the accurate dissecting plane during the entire hepatectomy, which, therefore, could provide a sound option for high‐precision, real‐time navigation of liver cancer surgery.

## 2 MATERIALS AND METHODS

### 2.1 Study design and patients

This study was approved by the Institutional Review Board and Ethics Committee of the Affiliated Zhong'shan Hospital of Xiamen University, Affiliated Hospital of Southwest Medical University, and Affiliated Hospital of North Sichuan Medical College in China. It is registered in the Chinese Clinical Trial Registry (Register No. ChiCTR2000035055).

A clinical application study was conducted to evaluate the usefulness and safety of SHIFT and ICG formulation prepared via SHIFT for surgical navigation after long‐term TAE. Between July of 2019 and April of 2022, 25 liver cancer patients who cannot be radically resected underwent TAE with SHIFT and ICG as the main embolic agent at the Affiliated Zhong'shan Hospital of Xiamen University, Affiliated Hospital of Southwest Medical University, and Affiliated Hospital of North Sichuan Medical College. We obtained consent from all patients, regardless of gender or age. The clinical data of 20 liver cancer patients who received TAE with conventional lipiodol as the main embolic agent were used as a control group. We conducted a retrospective analysis of the clinicopathological data to compare outcomes between the two groups.

### 2.2 Inclusion and exclusion criteria

Inclusion criteria: (1) patients with liver cancer and high surgical potential, confirmed by multiple imaging examinations (ultrasound, enhanced computerized tomography [CT], or magnetic resonance imaging [MRI]) or histological or cytological diagnosis, regardless of gender or age with an expected lifespan of ≧3 months; (2) intrahepatic tumors with single or multiple lesions located in half of the liver or confined to three adjacent liver lobes, with at least one lobe of the liver having a primary portal vein branch that is unobstructed, and barcelona clinic liver cancer (BCLC) stage A or B^24^; (3) Child‐A liver function or Child‐B liver function that can reach Child‐A after treatment and nutritional supplementation; and (4) preoperative and postoperative advantages, disadvantages, and risks were informed to patients in detail. Patients and their family members chose the treatment plan voluntarily and signed the informed consent.

Exclusion criteria: (1) patients with recurrent disease or who were previously treated by surgery or drugs; (2) patients with other confirmed malignant tumors or serious diseases of the heart, lung, kidney, brain, and other important organs; and (3) those with an indocyanine green allergy or iodine allergy.

### 2.3 Materials

Lipiodol was purchased from Jiangsu Hengrui Pharmaceutical Co, Ltd, Jiangsu, China. Indocyanine green was purchased from Dandong Medical and Pharmaceutical Co, Ltd, Dandong, China. An anti‐Ki‐67 antibody, 4′,6‐diamidino‐2‐phenylindole (DAPI), and terminal deoxynucleotidyl transferase dUTP nick end labeling (TUNEL) reagent box were purchased from Servicebio.

### 2.4 Equipment

**FIGURE 2:**
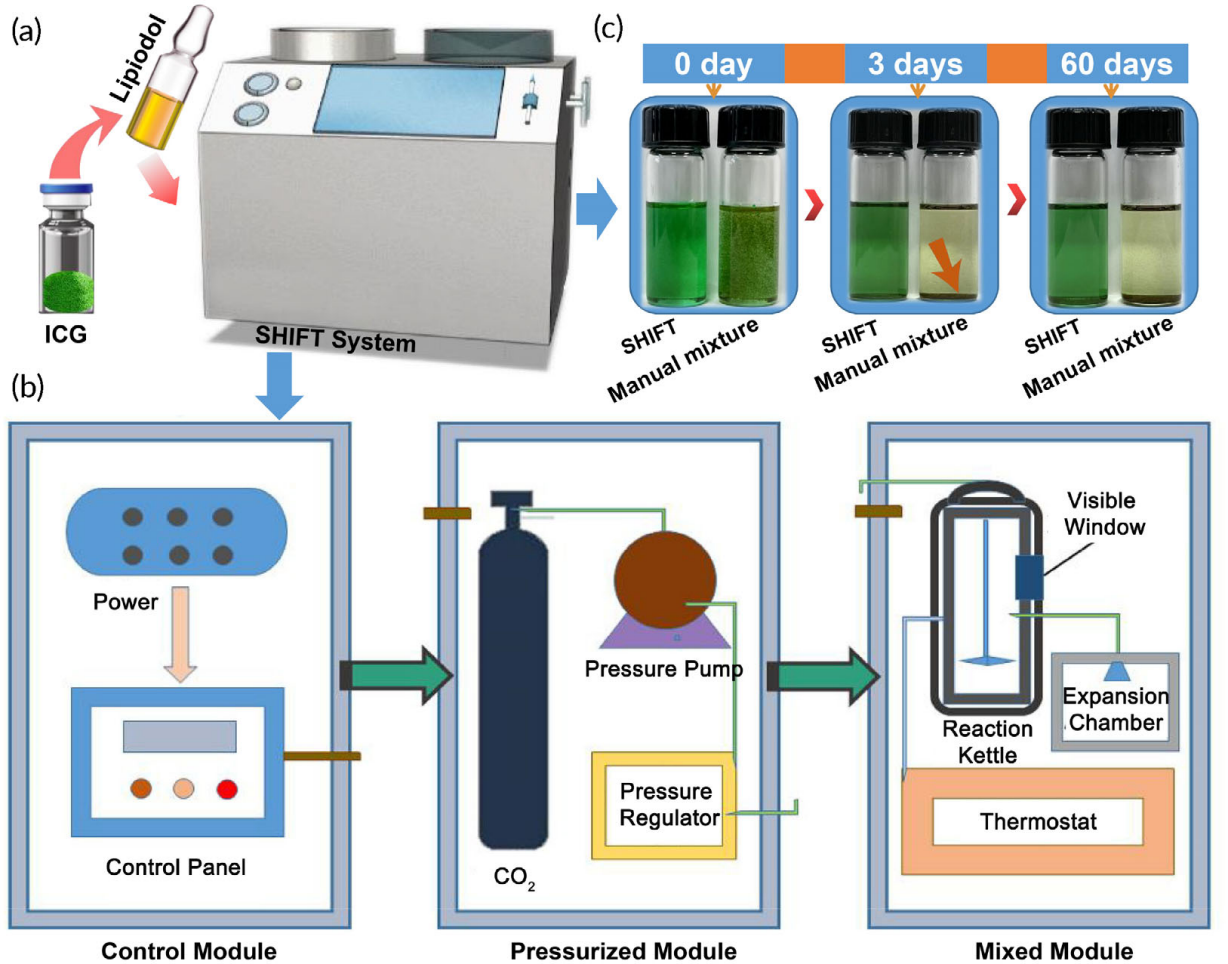
The super‐stable homogeneous intermix formulating technology (SHIFT) system. (a) A pictorial view of the SHIFT equipment, showing the miniature appearance and operation interface. (b) The module composition of the SHIFT equipment, including the control module, pressurized module, and drug mixed module. (c) The purpose of the SHIFT equipment is to achieve a stable and uniform formulation preparation of lipiodol and indocyanine green (ICG) drugs by supercritical carbon dioxide (CO_2_).

The fluorescent surgical navigation system (DPM‐01) was provided by the Key Laboratory of Molecular Imaging, Institute of Automation, Chinese Academy of Sciences, Beijing, and Beijing Digital Precision Medicine Technology Co. Ltd. The SHIFT equipment (Figure 2a) was developed by our laboratory (Patent nos. 2019107349374.4, 201910105683X, and 16581600), the Center for Molecular Imaging and Translational Medicine, School of Public Health, Xiamen University. The equipment consists of a control module, pressure module, and drug mixed module, which can realize the real‐time drug addition, preparation, collection, and equipment cleaning processes (Figure 2b), without any co‐solvents or toxic reagents to achieve a stable and uniform formulation preparation of lipiodol and ICG drugs.

The SHIFT and ICG formulation prepared by this method is only a mixture of lipiodol and ICG without chemical reactions or molecular changes in drugs.^21^ More importantly, it has excellent stability and homogeneity. An observation at room temperature determined that after 60 days, ICG molecule in SHIFT and ICG group was still clear and stably dispersed in lipiodol without delamination (Figure 2c).

### 2.5 Surgical methods

#### 2.5.1 Transarterial embolization and ICG administration

We punctured the femoral artery and performed celiac trunk and superior mesenteric artery angiography. After the tumor‐supplying artery was identified, a microcatheter was used to super‐selectively enter the target artery. First, local perfusion of the tumor was performed with lobaplatin for injection, and next an appropriate amount of SHIFT and ICG formulation (ICG concentration 1 mg/ml) (SHIFT and ICG group) or conventional lipiodol (control group) were injected respectively. The dosage of SHIFT and ICG formulation was injected according to the size of the tumor, generally within 10–20 ml,^25^ until target artery blood stasis or regurgitation occurs and the embolization stops.^26^ Finally, gelatine sponge particles (350–560 μm) sandwich method was used to strengthen embolization. Every 4 weeks follow‐up reexamination after TAE was conducted to clarify the treatment effect and the next treatment plan. The ICG was injected intravenously (*i.v*.) at a dose of 0.5 mg/kg 3–5 days before surgery after successful TAE in the control group.

#### 2.5.2 Fluorescence‐guided surgical resection

After successful tumor conversion therapy was confirmed by reexamination of CT or MRI and reached the standard of radical surgical resection, the resection of the liver tumor was performed under real‐time fluorescence navigation. In this study, all liver tumors and benign lesions were observed by intraoperative ultrasonography (IOUS) and fluorescent surgical navigation system, respectively. First, the lesion location, number, characteristics, and relationship to the surrounding structure were determined on the surface of the whole liver with the preoperative imaging examination and IOUS system. Then the fluorescence surgery navigation system was applied to lighten the liver to observe lesions from a distance of 5–20 cm above the liver surface. The contrast mode of the equipment was used to ensure a boundary between tumor and normal liver tissue. Afterward, the tumor lesions could be resected under the guidance of the lit area on the screen. Subsequently, the resected tissues were investigated by a fluorescence surgery navigation system to verify the ICG signal and distribution. Finally, H&E and immunohistochemistry staining were utilized to evaluate the resection and tumor tissues. Meanwhile to evaluate the SHIFT and ICG fluorescent navigation effect and resection quality, fluorescent signal area and intensity of the tumor sites and the signal‐to‐normal tissues ratio (SNR) were calculated using Image J and regions of interest (ROIs).

### 2.6 Statistical analysis

Data analyzed by Prism 7 (GraphPad Software, San Diego, California) and SPSS 17.0 were presented as mean ± SD. The Mann–Whitney U test was used to evaluate the differences between the experimental group and the control group. *p* values < 0.05 were considered statistically significant. *p < 0.05; **p < 0.01; ***p < 0.001; ****p < 0.0001.

## 3 RESULTS

### 3.1 General information and baseline characteristics

In this study, 45 patients with unresectable liver cancer, including 29 men and 16 women, with a mean (±standard deviation) age of 57.05 ± 10.27 and 54.8 ± 12.14 years in the control and SHIFT and ICG group, respectively, were successfully treated with TAE, using conventional lipiodol or SHIFT and ICG formulation as the main embolization agents. An irregular liver surface due to cirrhosis was found in 16 patients. The mean ICG‐R15 was 7.17% ± 2.58% and 7.28% ± 2.24% in the control and SHIFT and ICG group, respectively. No intraoperative or postoperative adverse events were observed related to the administration of ICG. The mean tumor size was 9.63 ± 2.35 cm and 9.18 ± 2.06 cm in the control and SHIFT and ICG group, respectively. The basic characteristics and demographic information of both experimental groups are shown in Table 1, with no significant between‐group differences identified.

**TABLE 1:** Patient characteristics and pathological variables

Patient characteristics	Control group (*n* = 20)	SHIFT and ICG (*n* = 25)	*p* value
Median age (Mean ± SD)	57.05 ± 10.27	54.80 ± 12.14	0.411
Male/female (*n*)	13/7	16/9	
Tumor location			
s2/s3/s4/s5/s6/s7/s8	1/0/4/5/6/1/3	2/0/7/5/6/2/3	
Background liver status			
Normal/chronic hepatitis/cirrhosis	4/9/7	6/10/9	
Child‐Pugh score			
A/B/C	17/3/0	18/7/0	
ICG R15 (% [Mean ± SD])	7.17 ± 2.58	7.28 ± 2.24	0.871
BCLC stage			
A/B/C/D	6/11/3/0	16/9/0/0	
Median tumor number (Mean ± SD)	1.35 ± 0.67	1.28 ± 0.54	0.855
Median size of largest tumor (cm) (range)	9.63 ± 2.35	9.18 ± 2.06	0.631

### 3.2 Evaluation of embolization performance and safety of the SHIFT and ICG formulation in patients with unresectable liver cancer

**FIGURE 3:**
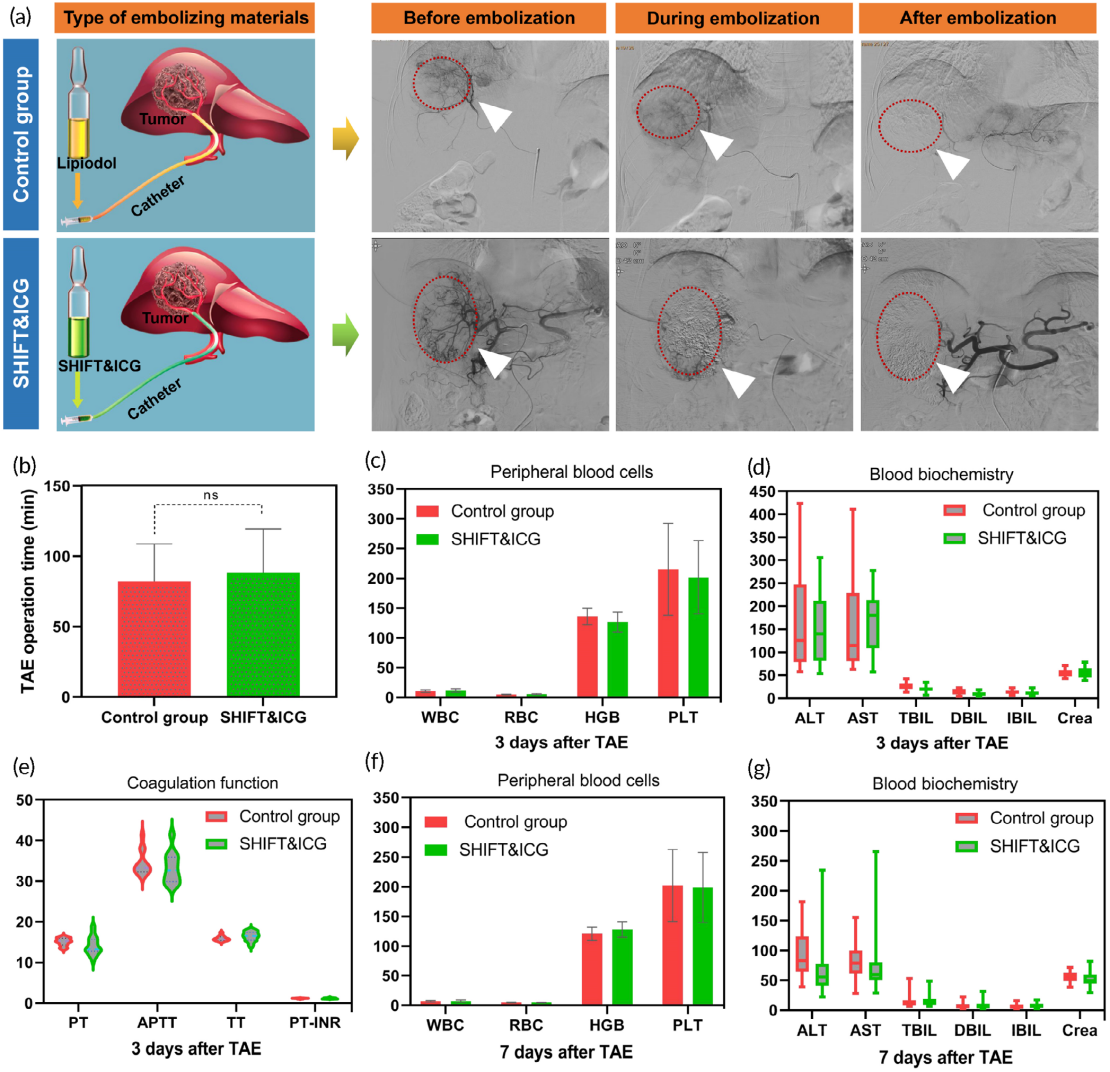
Evaluation of embolization performance and safety of super‐stable homogeneous intermix formulating technology (SHIFT) and indocyanine green (ICG). (a) The embolization process and deposition effect of traditional lipiodol and SHIFT and ICG formulation via DSA navigation. (b) The comparison of transcatheter arterial embolization (TAE) operation time. (c–e) The comparison of peripheral blood cells (c), biochemical examination (d), and blood coagulation (e) at 3 days post‐TAE. (f, g) The comparison of peripheral blood cells (f) and biochemical examination (g) at 7 days post‐TAE

Maintaining viscosity performance and safety are crucial for effective embolization. Through the TAE treatment of 45 liver cancer patients who could be radically resected, we found that both the SHIFT and ICG formulation and conventional lipiodol group all deposited well in the liver cancer lesions during and after embolization (Figure 3a). No significant difference was found in TAE operation duration between the two groups (82.00 ± 26.70 vs. 88.32 ± 30.93 min; *p* = 0.486; Figure 3b). The peripheral blood cells, biochemical examination, and blood coagulation results of the two groups were collected on the third and seventh days post‐TAE.

No obvious abnormality was found in the peripheral blood cells and blood coagulation indices of the two groups (Figure 3c,e,f). Although alanine transaminase (ALT) and aspartate transaminase (AST) increased slightly on the third day after TAE in both groups, they returned nearly to normal on the seventh day (Figure 3d,g). This indicated that the SHIFT is only a physical process and does not alter the embolization performance of SHIFT and ICG. As a major embolization agent, SHIFT and ICG has an excellent safety profile.

### 3.3 Evaluating the performance of fluorescence‐guided precision surgery

#### 3.3.1 The surgical navigation effect of ICG injection 3–5 days before surgery after successful conversion therapy in the control group

**FIGURE 4:**
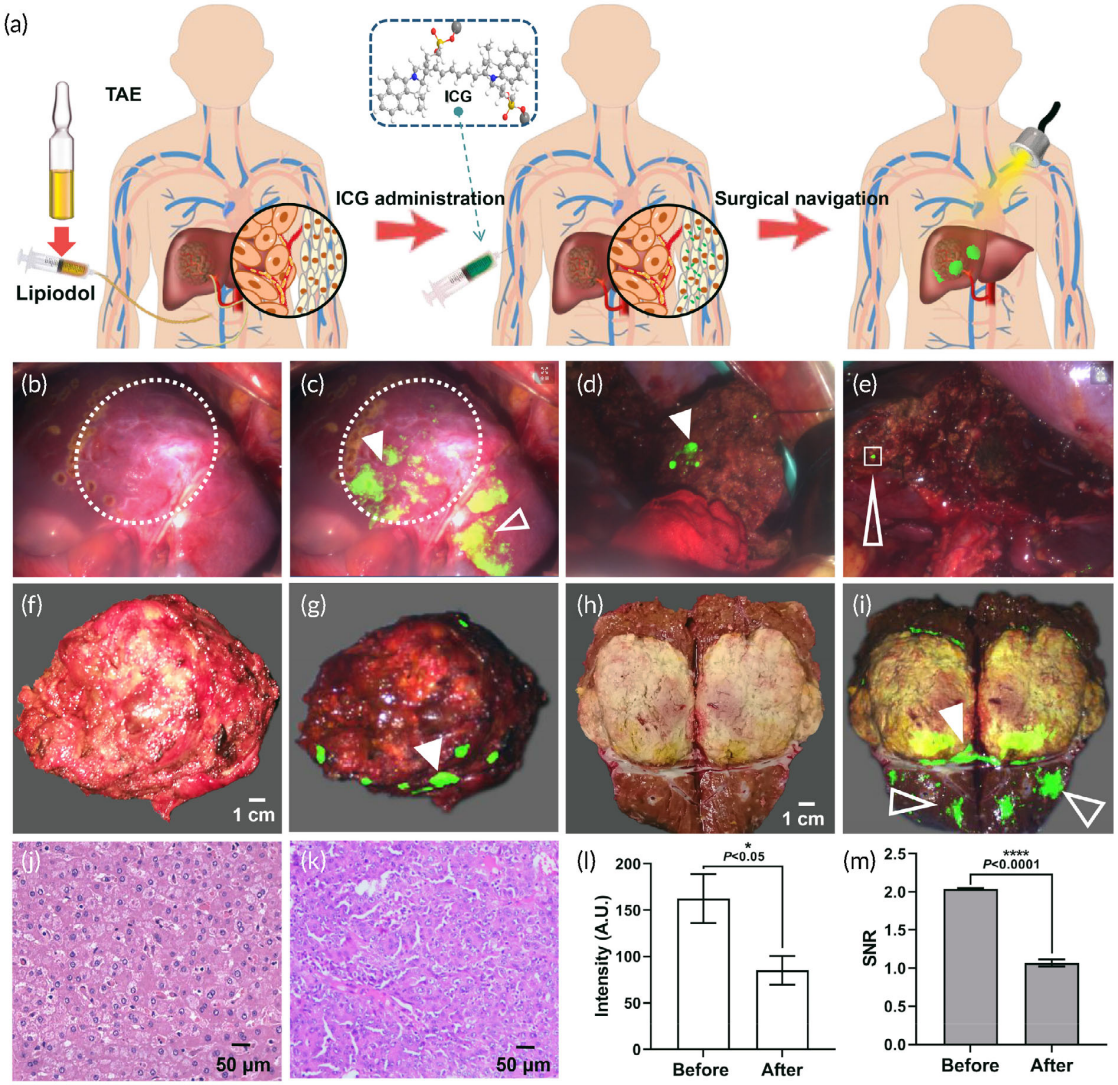
The representative surgical navigation effect of indocyanine green (ICG) injected 3–5 days before surgery after successful conversion therapy. (a) The schematic illustration of traditional ICG injected intravenously after successful conversion therapy with poor surgical navigation effect. (b, c) Liver cancer lesion (b, dotted white circle) and fluorescence imaging (c, solid white arrowhead: tumor lesions, blank white arrowhead: normal tissues). (d) The image of intraoperative real‐time fluorescence (solid white arrowhead). (e) Fluorescence imaging of the incisal margin of the residual liver and normal liver fluorescence tissue (blank white arrowhead). (f, g) Whole resected tumor lesions (f) and fluorescence imaging (g, solid white arrowhead). (h, i), Cut tumor lesions (h) and fluorescence imaging (i, solid white arrowhead: tumor lesions, blank white arrowhead: normal tissues). (j, k) The H&E pathological examination of resected tissues (j, residual fluorescence signals tissue confirmed normal liver tissue; k, resected tumor tissue). (l, m) The fluorescence signal intensity and SNR of tumor site before and after resection. A.U., “arbitrary unit”

In the control group, conversion therapy was successful in three patients and reached the standard of radical surgical resection. ICG was then injected i.v. 3–5 days before the operation. Although the tumor could be located before resection, its location was incomplete, and only a few weak fluorescence signals were seen at the tumor lesions (Figure 4a). The boundary between the tumor and normal tissues could not be distinguished accurately (Figure 4b,c, white dotted circle). A large number of fluorescence signals were observed beyond the actual boundary of the tumor (Figure 4c, white blank arrowhead). The real‐time navigation of tumor resection indicated only a few weak fluorescence signals in the tumor lesions (Figure 4d). Moreover, fluorescence imaging of the remaining liver sections showed residual fluorescence signals, which were confirmed by H&E pathological examination to be normal liver tissue (Figure 4e,j).

To further clarify the effect of surgical navigation by *i.v*. injection of ICG, we performed fluorescence imaging of the lesions and transected the surfaces of resected tumor lesions. The results showed that the whole resected tumor lesions showed only minimal fluorescence signals, and the tumor boundary could not be distinguished (Figure 4f,g). For the resected tumor lesions, small fluorescence signals were located in liver tumor tissues (Figure 4h,i, white solid arrowhead), while most of the fluorescence signals were observed in the normal liver tissues (Figure 4h,i, white blank arrowhead). As shown in Figure 4k, the boundary between the tumor and normal liver tissue was not indicated, resulting in the removal of a large portion of normal liver tissues. Quantitative evaluation results of tumor resection quality showed that, prior to the resection, the fluorescence intensity and signal‐to‐noise ratio of each tumor site was 162.62 ± 26.52 and 2.04 ± 0.01, respectively. After resection, the fluorescence signal intensity of the tumor sites reduced to 85.10 ± 15.56, and the decreased SNR (1.07 ± 0.05) was close to 0.97 (Figure 4l,m). These results suggest that after successful conversion therapy, the traditional *i.v*. ICG method is unable to provide effective and accurate navigation of surgical excision for liver cancer.

#### 3.3.2 The surgical navigation effect of SHIFT and ICG

**FIGURE 5:**
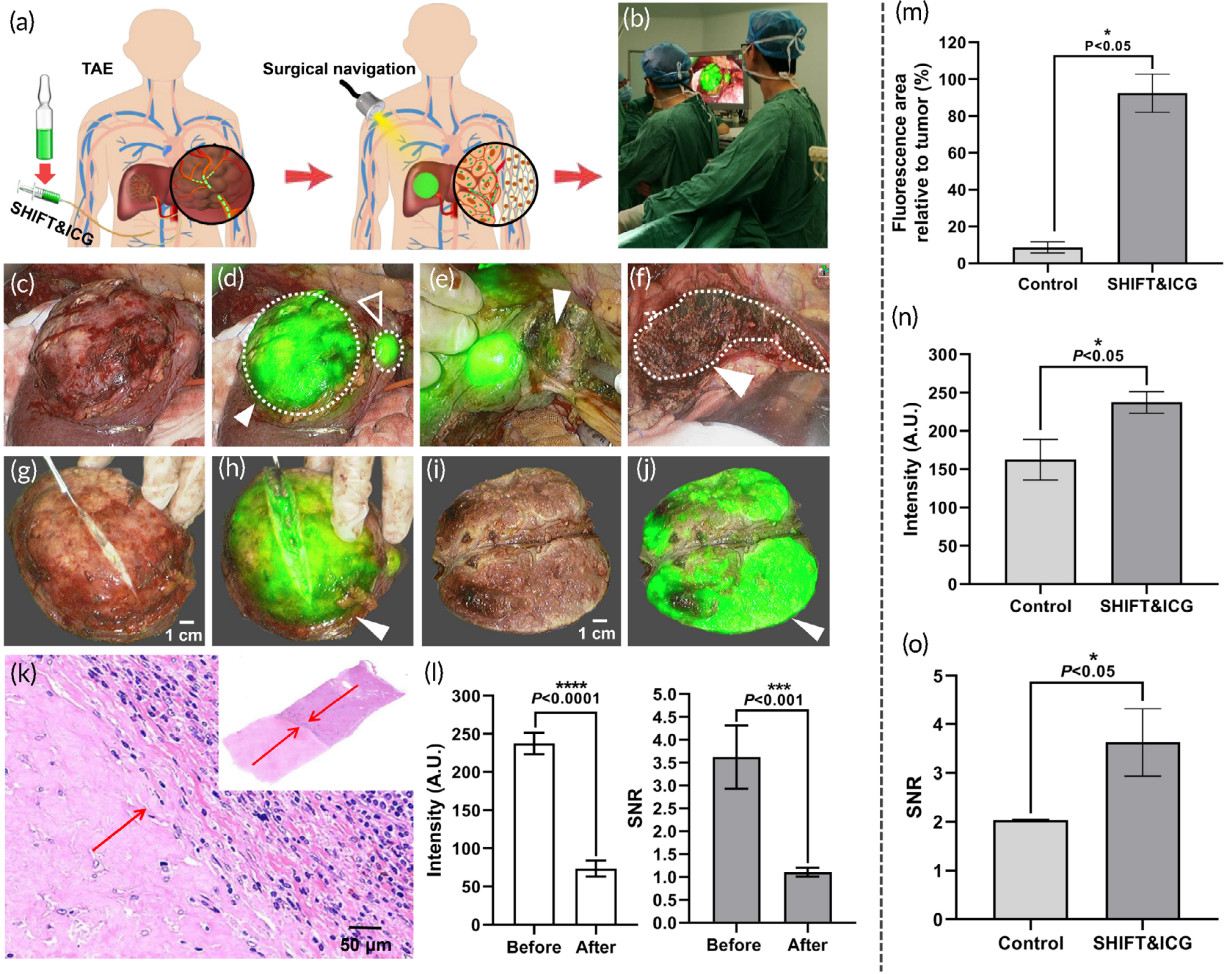
The representative surgical navigation effect of super‐stable homogeneous intermix formulating technology (SHIFT) and indocyanine green (ICG) after successful conversion therapy. (a), Schematic illustration of SHIFT and ICG after successful conversion therapy, with excellent surgical navigation effect. (b) Photograph of the intraoperative view. (c, d) Liver cancer lesions and fluorescence imaging (d, solid white arrowhead: primary tumor lesions about 8 × 11 cm^2^ in size; blank white arrowhead: tiny satellite tumor lesions about 1.5 × 2 cm^2^ in size). (e) Intraoperative real‐time fluorescence‐guided tumor resection (solid white arrowhead). (f) Fluorescence imaging of the incisal margin of the residual liver, with achieved R0 resection (solid white arrowhead). (g, h) Whole resected tumor lesions (g) and fluorescence imaging (h, solid white arrowhead). (i, j) Cut tumor lesions (i) and fluorescence imaging (j, solid white arrowhead). (k) Pathological examination of the resected tissues, with a clear boundary seen between the tumor and normal tissue (red arrow). (l) Fluorescence signal intensity and SNR of the tumor site before and after resection. (m–o) Fluorescence area relative to tumor (m), intensity (n), and SNB of tumor site (o) before resection for both groups. A.U., arbitrary unit

Conversion therapy was successful in four patients treated with SHIFT and ICG for TAE, reaching the standard of radical surgical resection, as confirmed by CT (Figure S1). Surgical resection under real‐time fluorescence navigation was performed on these four patients. The CT imaging confirmed that SHIFT and ICG was fully deposited in the tumor area (Figure S1). Subsequently, we further verified that the SHIFT and ICG could exert fluorescent navigation function after embolization (Figure 5a,b). The results indicated that SHIFT and ICG exhibited a superior fluorescent capability compared to controls, and before the tumor was resected, bright fluorescent signals were evenly distributed throughout the tumor lesions. The entire tumor lesions were illuminated (Figure 5c,d, dotted white arrowhead), and the boundary between the tumor and normal liver tissues was clearly visualized (Figure 5d, dotted white circle). More importantly, the minuscule satellite tumor lesions surrounding the primary tumor were also visualized (Figure 5d, blank white arrowhead), establishing excellent conditions for guiding hepatectomy. In the real‐time navigation of tumor resection, fluorescence signals are emitted from large portions of the tumor lesions (Figures 5e, S2). This could indicate the boundary between the liver tumor and normal liver tissues to guide a precise surgical resection. After tumor was resected, the remaining liver sections were neat, clean, and devoid of residual fluorescent signals (Figure 5f, dotted white circle). Meanwhile, postoperative CT examination also indicated no residual tumor tissue, reaching R0 resection (Figure S3).

To further clarify the effect of surgical navigation based on SHIFT and ICG, we performed fluorescence imaging of the entire tumor lesions and the transected surfaces of the resected tumor lesions. The results showed that the whole lesions were illuminated by the fluorescent signals of SHIFT and ICG, allowing the tumor boundary to be accurately distinguished (Figure 5g,h, solid white arrowhead). For the tumor lesions that were cut open, the fluorescent signal illuminated the entirety of the cut surfaces, with no fluorescent signal observed in normal liver tissues. The boundary delineating the tumor from normal liver tissues was clear, reaching the R0 resection (Figure 5i,j, solid white arrowhead), which was confirmed by postoperative pathological H&E staining (Figure 5k). Only a small amount of normal liver tissues was excised beyond the tumor boundary, which suggests that SHIFT and ICG has ultra‐high sensitivity for guiding a precise hepatectomy and for reserving normal liver tissue for a good prognosis.

The quantitative evaluation results of tumor resection quality showed that, prior to resection, the fluorescence intensity and SNR of each tumor site was 237.55 ± 14.14 and 3.63 ± 0.69, respectively. After resection, the fluorescence signal intensity of the tumor sites decreased to 73.76 ± 10.33, and the decreased SNR (1.11 ± 0.10) neared 2.52 (Figure 5l). Quality evaluation of the fluorescence navigation before resection for both groups indicated that the fluorescence area relative to the tumor of patients in the SHIFT and ICG group was significantly larger than that of controls (92.5 ± 10.38 vs. 8.67 ± 3.06, *p* = 0.032, Figure 5m), and both the fluorescence intensity (237.55 ± 14.14 vs. 162.62 ± 26.52, *p* 0.034, Figure 5n) and SNB (3.63 ± 0.69 vs. 2.04 ± 0.01, *p* = 0.034, Figure 5o) were significantly higher than those of the control group. The significant differences (*p* < 0.05) in the quantitative results indicated that SHIFT and ICG can guide liver tumor resection more effectively than previously available methods.

To further verify the fluorescence imaging and embolization effect of SHIFT and ICG, we performed an immunohistochemical examination of resected tumor specimens with Ki‐67, TUNEL, and DAPI. The results showed many ICG fluorescence signals in the tumor tissues, a low expression level of Ki‐67, and a high expression level of TUNEL (Figure S4), indicating that SHIFT and ICG, through TAE, is capable of achieving tumor embolization and tissue necrosis while visualizing the tumor.

### 3.4 Comparison of operative, anesthetic, and postoperative variables

**FIGURE 6:**
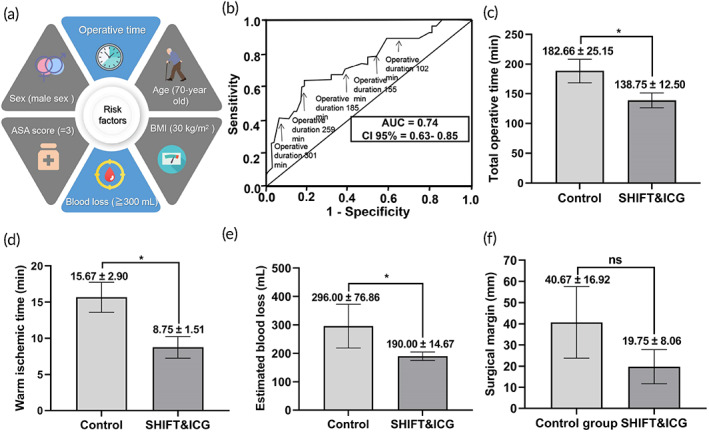
The comparison of operative and anesthetic variables. (a) The major risk factors for postoperative complications. (b) Receiver‐operating characteristic (ROC) curve, revealing the impact of the operative duration on postoperative complication risk. AUC, area under the curve; CI, confidence interval.^27^ (c–f) The comparison of total operative time (c), warm ischemic time (d), estimated blood loss (e), and surgical margin (f)

Postoperative complications can affect prognosis and survival rate. The major risk factors for postoperative complications include the male sex, advanced age (≥70 years old, obesity (BMI = 30 kg/m^2^), ASA score (3), operative duration, and blood loss ≧300 ml; Figure 6a). Total operative duration (OR = 1.008 [1.003–1.01]; *p* = 0.001) was a key independent predictor of postoperative morbidity (Figure 6b). The postoperative complication rate increases by 60% with each additional operative hour during liver resection.^27^ Therefore, minimizing the total operative time and blood loss is crucial for preventing serious postoperative complications and for improving prognosis. An analysis of operative, anesthetic, and postoperative variables of patients undergoing liver resection found that the total operative time (182.66 ± 25.15 vs. 138.75 ± 12.50 min; *p* = 0.034; Figure 6c) and warm ischemic time (15.67 ± 2.089 vs. 8.75 ± 1.51 min; *p* = 0.032) in the SHIFT and ICG group were shorter (Figure 6d), and the estimated blood loss (296.00 ± 76.86 vs. 190.00 ± 14.67 ml; *p* = 0.034) was lower (Figure 6e), compared to the control group (Table S1).

These results suggest that SHIFT and ICG is capable of rapid and accurate tumor identification and of reducing the risk of complications after hepatectomy. Although R0 resection was achieved in both groups, the surgical margin in the SHIFT and ICG group (19.75 ± 8.06 mm) was significantly smaller than that in the control group (40.67 ± 16.92 mm; *p* = 0.157; Figure 6f). Furthermore, analysis of the postoperative morbidity and mortality variables in both groups showed that one patient in the control group developed ascites, while no obvious abnormalities were found in the SHIFT and ICG group. Additionally, no deaths were reported in either group at 30 and 60 days post‐surgery (Table S2).

## 4 DISCUSSION

Liver cancer, which constitutes the most common tumor of the digestive system, is characterized by rapid onset, a high degree of malignancy, and rapid invasive growth; it is usually diagnosed in its middle and advanced stages.^28^, ^29^ Surgical resection is the first line of treatment^30^; however, many patients are diagnosed too late for surgery, or when the tumor volume is too large. In these cases, direct resection can cause great trauma or residual tumor tissues with poor prognosis. These patients can be treated with secondary surgical resection after TAE or transcatheter arterial chemo‐embolization of conversion therapy.^31^, ^32^ Unfortunately, identifying the boundary that delineates the tumor from normal liver tissue and microscopic lesions is difficult during surgical resection. Cancerous tissue often cannot be removed completely, leading to postoperative tumor recurrence.^33^, ^34^ Therefore, the development of an effective fluorescent molecular probe is urgently needed to distinguish the boundary between tumors and both normal tissue and microscopic lesions.^12^, ^35^ In clinical practice, lipiodol is a common agent in interventional embolization for liver cancer, due to its high viscosity and precise tumoral deposition.^36^ However, sufficient delivery of the fluorescent probe into the tumor region after interventional embolization can prove challenging. Thus, we here present a strategy using a prepared fluorescent probe and lipiodol formulation for precise fluorescent surgical navigation after long‐term TAE conversion therapy.

The ICG molecule is quickly metabolized, has photobleaching resistance and no targeting ability, and is insoluble in oily substances.^37^ If fluorescence navigation is performed by traditional *i.v*. injection after TAE treatment, it is highly difficult for ICG delivery in cancer tissues because the feeding artery of liver cancer is embolized. In this study, the simple, green, and economical SHIFT technology alleviated the problems associated with the ICG molecule and, thereby, overcame the barriers presented by traditional methods. We constructed SHIFT technology using a physical method to stably disperse ICG molecules in lipiodol for superior conditions for fluorescent surgery after long‐term TAE of conversion therapy. Our previous experimental results showed that SHIFT and ICG was sufficiently stable to protect the fluorescence performance of ICG in vitro. More strikingly, SHIFT and ICG could serve as a good embolic agent and may block the tumor progression of VX2 orthotopic hepatocellular carcinoma models.^21^, ^22^, ^23^ This clinical experiment proved that SHIFT and ICG, like traditional lipiodol, had excellent specific tumor deposition in TAE treatment. The formulation did not affect the process and duration of the TAE operation. More importantly, the formulation had the same excellent safety and tumor embolization effect as traditional lipiodol. This pure physical SHIFT technology is safe and reliable and could be used in clinical diagnosis and treatment.

Furthermore, the ability of ICG fluorescence to locate tumor lesions is key for the accurate resection of navigation surgery and has important clinical implications. Our results showed that when ICG was injected i.v. after TAE of conversion therapy, the ICG fluorescence signals were only weakly distributed in tumor tissues, while most signals remained in normal liver tissues. This could not accurately identify tumor lesions and boundaries, resulting in the majority of normal liver tissue being removed. After TAE, the tumor‐supplying artery was embolized, and ICG molecules were unable to enter the tumor tissue, which may explain the procedure's poor outcomes. Although the rapid plasma clearance, unstable fluorescence feature, and relatively low ICG concentration may also play a role,^18^ high ICG concentrations (up to 0.5 mg/kg per patient) cause side‐effects including fever, shock, and allergy.^38^ The excellent fluorescence intensity, SNR, and wide fluorescence area afforded by SHIFT and ICG has better optical properties relative to i.v.‐free ICG. Lipiodol has excellent specific tumoral deposition and allows for precise and enduring guided hepatectomy; notably, it clearly illuminated the heretofore indiscoverable boundary between the tumor and healthy tissue and satellite lesions of tumors, and it could remove tumor tissue while retaining normal liver tissue, thereby contributing to preventing postoperative liver failure.

Moreover, SHIFT and ICG could shorten the total operation duration and warm ischemic duration, reduce intraoperative bleeding and trauma, and allow for more accurate surgery and more precise tumor resection. These features would support rapid postoperative recovery, due to minor trauma and fewer risk factors.^39^, ^40^ The rapid and accurate identification of tumors by fluorescence navigation technology guides surgeons to remove tumors quickly and simplifies the complicated process of traditional surgery. In brief, SHIFT and ICG is an effective, green, and safe fluorescent‐guided formulation for precise surgical navigation. It overcomes the deficiency of traditional fluorescent navigation after interventional embolization and is valuable in clinical diagnosis and treatment. Taken together, SHIFT and ICG may act as a novel and safe agent capable of effectively transforming advanced liver cancer and of achieving accurate resection of liver cancer.

Nevertheless, our work has some limitations, such as a small sample size, early staged patients, a rich blood supply to the tumor, and a lack of long‐term outcomes data. These issues can be addressed by expanding the sample size, altering the inclusion criteria, and including close long‐term follow‐up; our future research aims to solve these issues. Furthermore, the SHIFT and ICG formulation can be extended to accurate, preoperative TAE‐assisted fluorescence navigation for radical resection of large or small liver cancer tumors. The SHIFT drugs can be extended to chemotherapeutics and radiopharmaceuticals to develop enhanced chemotherapy and radiotherapy.^41^, ^42^

## 5 CONCLUSIONS

In summary, such a green SHIFT and ICG formulation via SHIFT integrates the excellent stability, antiphotobleaching ability, imaging sensitivity, and specific tumoral deposition of lipiodol to address the clinical issues of fluorescent surgical navigation after long‐term embolization therapy of conversion therapy.

## AUTHOR CONTRIBUTIONS

**Pan He:** Data curation (equal); formal analysis (lead); funding acquisition (supporting); methodology (equal); visualization (equal); writing – original draft (lead). **Yongfu Xiong:** Data curation (equal); formal analysis (equal); methodology (equal); visualization (equal); writing – original draft (equal). **Bin Luo:** Data curation (equal); formal analysis (equal); methodology (equal); visualization (equal); writing – original draft (equal). **Jianming Liu:** Data curation (equal); formal analysis (equal); funding acquisition (equal); methodology (equal); visualization (equal); writing – original draft (equal). **Yang Zhang:** Conceptualization (equal); formal analysis (equal); methodology (equal); visualization (equal). **Yu Xiong:** Data curation (equal); formal analysis (equal); methodology (equal); visualization (equal). **Song Su:** Data curation (equal); methodology (equal); visualization (equal). **Cheng Fang:** Data curation (equal); methodology (equal); visualization (equal). **Yisheng Peng:** Data curation (equal); methodology (equal); visualization (equal). **Hongwei Cheng:** Formal analysis (equal); methodology (equal); visualization (equal). **Chengchao Chu:** Formal analysis (equal); methodology (equal). **Jingsong Mao:** Formal analysis (equal); methodology (equal). **Jingdong Li:** Conceptualization (equal); data curation (equal); formal analysis (equal); methodology (equal); visualization (equal); writing – review and editing (equal). **Gang Liu:** Conceptualization (lead); data curation (equal); formal analysis (lead); funding acquisition (lead); methodology (lead); project administration (lead); writing – review and editing (lead). **Bo Li:** Data curation (equal); formal analysis (equal); methodology (equal); visualization (equal); writing – review and editing (equal). **Zhenyu Yin:** Conceptualization (equal); data curation (equal); methodology (equal); visualization (equal); writing – review and editing (equal). **Jie Tian:** Conceptualization (equal); data curation (equal); methodology (equal); project administration (equal); visualization (equal); writing – review and editing (equal).

## CONFLICT OF INTEREST

The authors declare no competing interests.

## Supporting information


**Appendix S1** Supporting Information

## Data Availability

The data that support the findings of this study are available from the corresponding author upon reasonable request.
