# Supplementary material for: An exploratory human study of superstable homogeneous lipiodol–indocyanine green formulation for precise surgical navigation in liver cancer
Source: Bioeng Transl Med. 2022 Sep 10;8(2):e10404. doi: 10.1002/btm2.10404 (PMC10013747; doi:10.1002/btm2.10404)
Supplement: Supplementary file 1 — Appendix S1 Supporting Information [file BTM2-8-e10404-s001.doc]

**Supplementary Information**

**An exploratory human study of superstable homogeneous lipiodol-indocyanine green formulation for precise surgical navigation in liver cancer**

**Table of Contents:**

**-Supplementary Figures 1-4 and Tables 1-2**

**
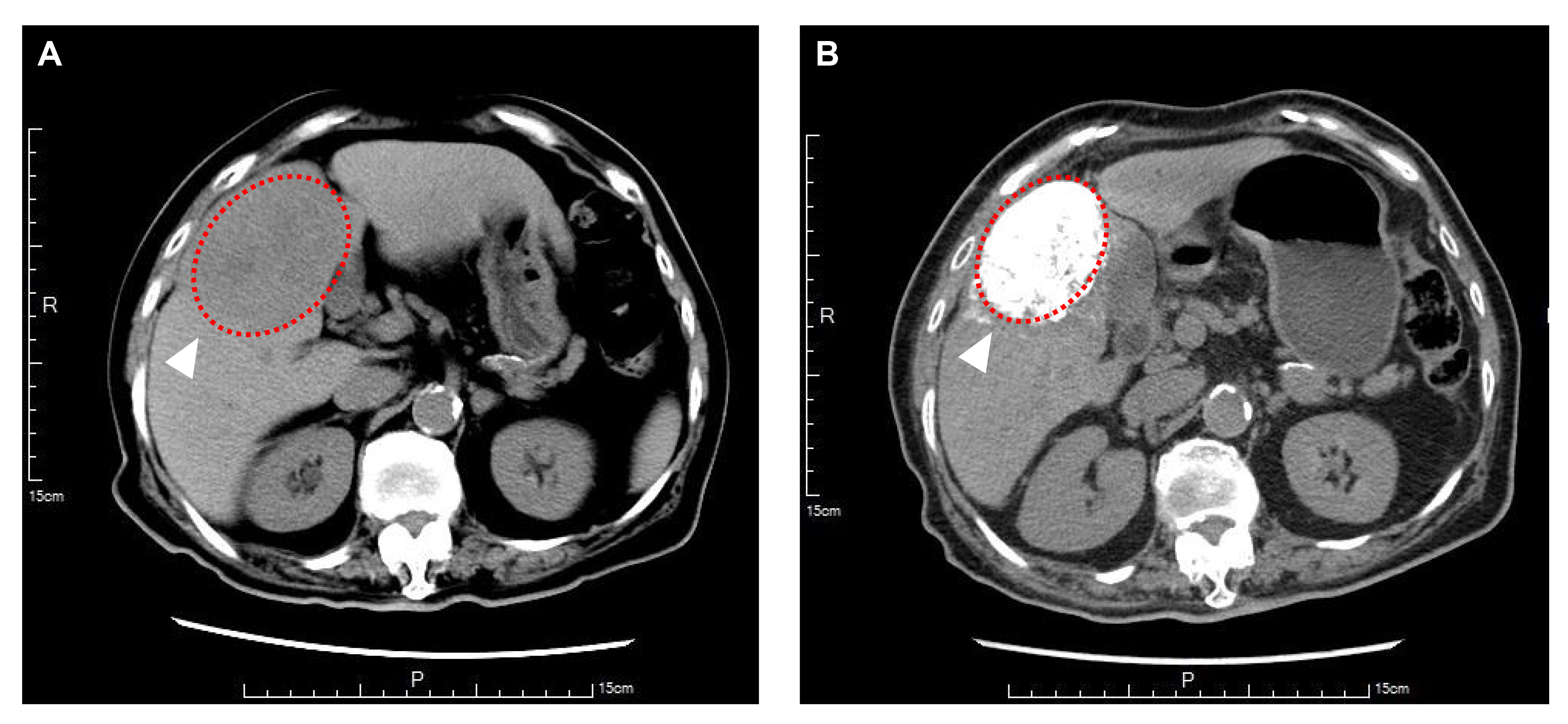
**

**Figure S1.** The computerized tomography (CT) image of representative patient. **A**, the CT examination at the first visit and before TAE. **B**, the CT examination after conversion therapy and before surgical resection.


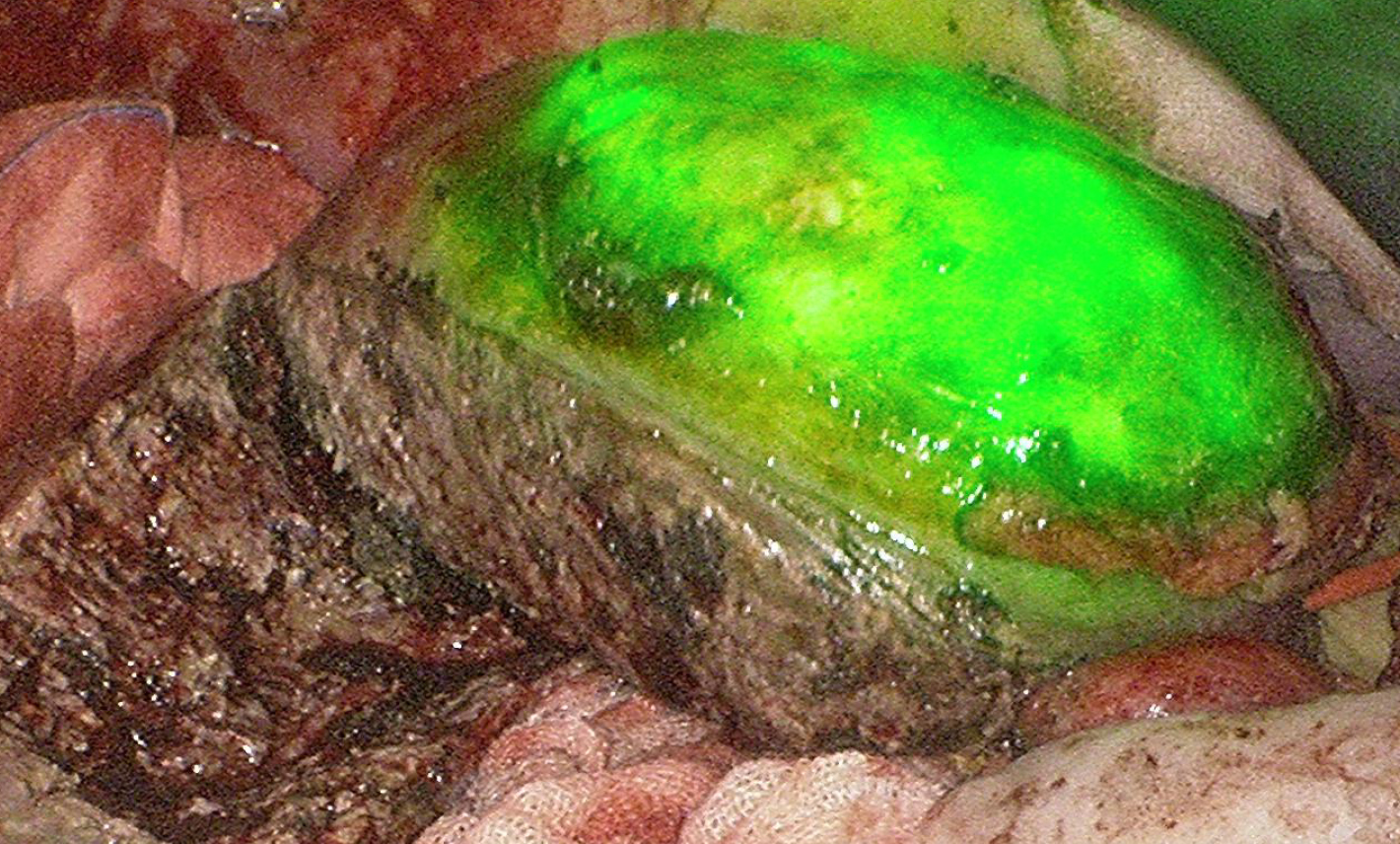


**Figure S2.** The image of intraoperative real-time fluorescence guided tumor resection.


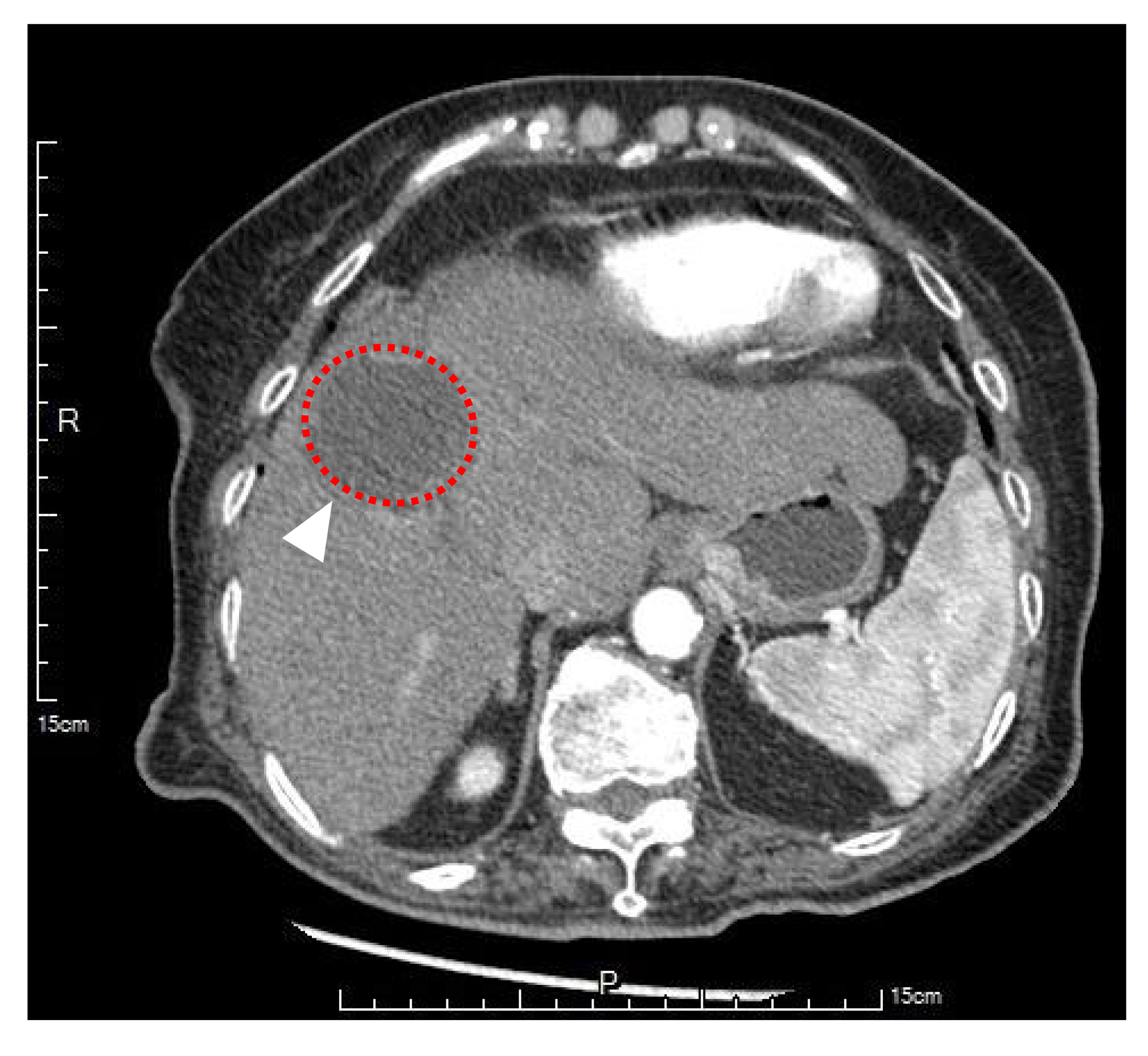


**Figure S3.** The CT examination after surgical resection, indicated no residual tumor tissue, reaching R0 resection.


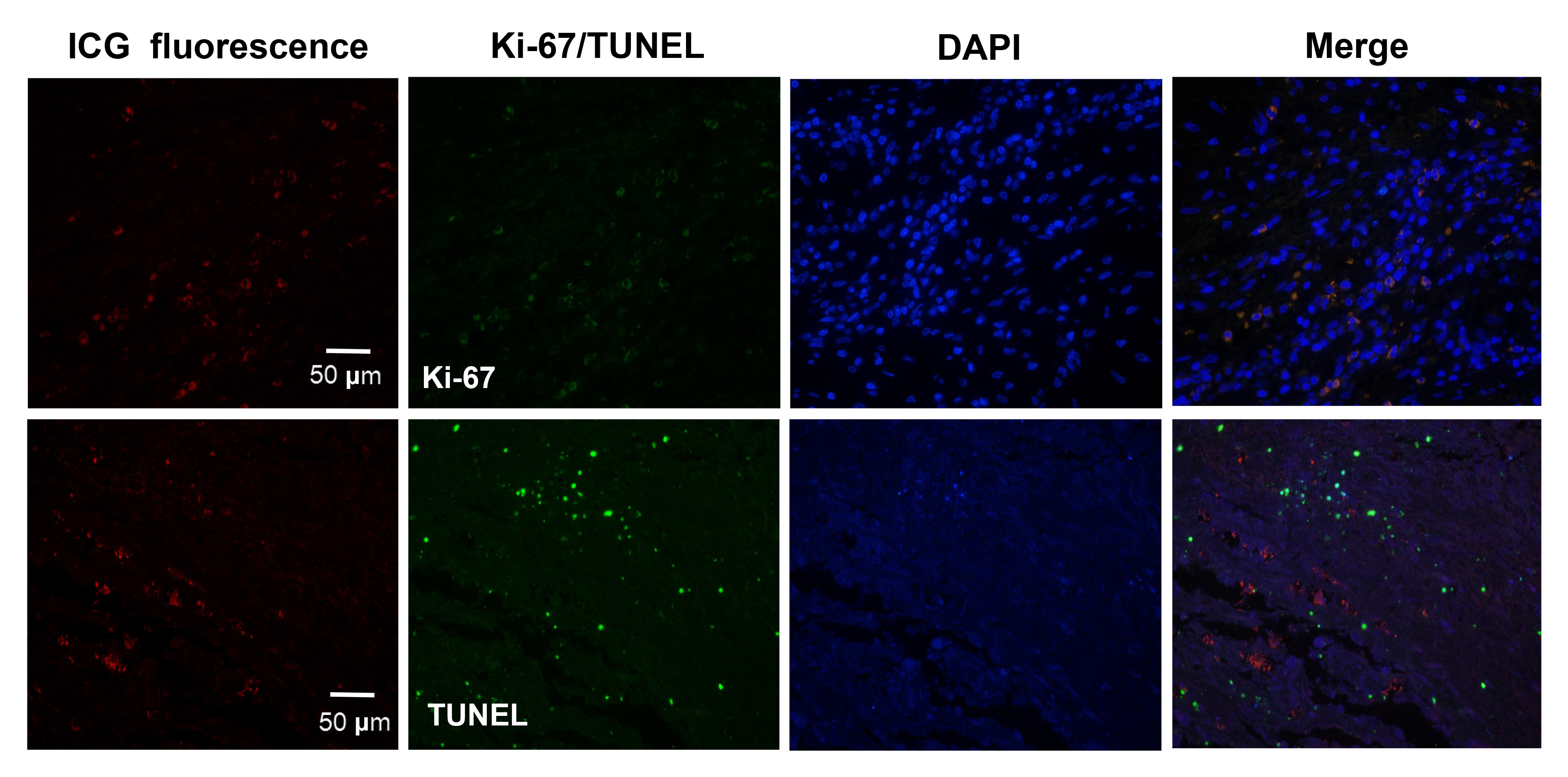


**Figure S4.** The immunofluorescence histological analysis, showed that there were a large number of ICG fluorescence signals in the tumor tissues, a low expression rate of Ki-67, and a high expression rate of TUNEL.

| **Table S1** Operative and anesthetic variables | | | |
| --- | --- | --- | --- |
| Variables | Control group (n = 20) | SHIFT&ICG (n = 25) | *P* value |
| TACE (n) | 20 | 25 |  |
| TACE operation time (min) | 82.00 ± 26.70 | 88.32 ± 30.93 | 0.486 |
| Surgical procedure |  |  |  |
| Limited resection | 3 | 4 |  |
| Total operative duration (min) |  |  |  |
| Mean ± SD | 182.66 ± 25.15 | 138.75 ± 12.50 | 0.034 |
| Warm ischemic duration (min) |  |  |  |
| Mean ± SD | 15.67 ± 2.089 | 8.75 ± 1.51 | 0.032 |
| Estimated blood loss (mL) |  |  |  |
| Mean ± SD | 296.00 ± 76.86 | 190.00 ± 14.67 | 0.034 |
| Surgical margin (mm) |  |  |  |
| Mean ± SD | 40.67 ± 16.92 | 19.75 ± 8.06 | 0.157 |
| Positive/Negative | 0/3 | 0/4 |  |

| **Table S2** Outcomes of patients undergoing liver resections | | |
| --- | --- | --- |
| Outcomes | Control group (n = 3) | SHIFT&ICG (n = 4) |
| Morbidity (Clavien-Dindo classification) |  |  |
| Biliary fistula | 0 | 0 |
| Postoperative bleeding | 0 | 0 |
| Pleural effusion | 0 | 0 |
| Ascites | 1 | 0 |
| Paresis of intestine | 0 | 0 |
| Liver failure | 0 | 0 |
| Other | 0 | 0 |
| Mortality (30 days/60 days) | 0/0 | 0/0 |
